# Epidemiology of *Streptococcus dysgalactiae* subsp. *equisimilis* in Tropical Communities, Northern Australia

**DOI:** 10.3201/eid1311.061258

**Published:** 2007-11

**Authors:** Malcolm McDonald, Rebecca J. Towers, Ross M. Andrews, Jonathan R. Carapetis, Bart J. Currie

**Affiliations:** * Menzies School of Health Research, Darwin, Northern Territory, Australia; † Royal Children’s Hospital, Melbourne, Victoria, Australia

**Keywords:** Streptococcus dysgalactiae subsp. equisimilis, group C streptococci, group G streptococci, S. pyogenes, emm sequence typing, Aboriginal Australian, pyoderma, pharyngitis, research

## Abstract

*Streptococcus dysgalactiae* subsp. *equisimilis* (groups C and G streptococci [GCS/GGS]) is an increasingly recognized human pathogen, although it may follow indirect pathways. Prospective surveillance of selected households in 3 remote Aboriginal communities in Australia provided 337 GCS/GGS isolates that were *emm* sequence-typed. Lancefield group C isolates (GCS) were localized to specific households and group G isolates (GGS) were more evenly distributed. GCS/GGS was more frequently recovered from the throat than group A streptococci (GAS [*S*. *pyogenes*]) but rarely recovered from skin sores, and then only with *Staphylococcus aureus* or GAS. Symptomatic GGS/GGC pharyngitis was also rare. Specific *emm* sequence types of GCS/GGS did not appear to cycle through the communities (sequential strain replacement) in a manner suggesting acquisition of type-specific immunity. These communities already have high levels of streptococcal and poststreptococcal disease. GCS/GGS may increase in importance as it acquires key virulence factors from GAS by lateral gene transfer.

## Abstract 2

This subspecies is common in communities with high rates of streptococcal disease, and its epidemiology differs from that of *Streptococcus pyogenes*.

In 1933, Rebecca Lancefield described a precipitin reaction that differentiated β-hemolytic streptococci into several groups according to the group-specific carbohydrate; these included groups A to E and unclassified strains (*1*). The isolates of group C streptococci (GCS) she investigated were of animal origin. Group G streptococci (GGS) were subsequently recognized in vaginal swabs from parturient women (*2*) and, for much of the next 50 years, GCS and GGS were considered to be nonpathogenic flora of the throat, gut, and vagina. When it became apparent that GCS and GGS could be human pathogens, it also emerged that they were a diverse group of streptococci consisting of at least 4 species, *Streptococcus anginosus*, *S*. *equi*, *S*. *equisimilis*, *and S*. *zooepidemicus* (*3*). In contrast, with few exceptions, group A streptococci (GAS) belong to 1 species, *S*. *pyogenes*.

Certain strains of GCS and GGS have been increasingly reported to cause infections similar to those caused by GAS such as pharyngitis, sepsis, skin and soft tissue infection, toxic shock, reactive arthritis, and postinfectious glomerulonephritis (*3*). Similar to GAS, human strains of GCS and GGS tend to have large colonies and a hyaluronic acid capsule; they also produce M protein that has structural, immunochemical, and biologic features similar to the M protein of GAS (*4*). Subsequent studies of the bacterial genome, including multilocus sequence typing of housekeeping genes, has demonstrated that large colony–forming human GCS and GGS are members of 1 species, *S*. *dysgalactiae* subsp. *equisimilis* (GCS/GGS) (*5*).

As with GAS, the M protein of GCS/GGS is responsible for resistance to phagocytosis (*4*). There is extensive sequence homology between gene sequences of GCS/GGS M protein and the *emm* gene of GAS; sequence heterogeneity at the 5′ end results in distinct *emm* sequence types (*6*). More than 35 years ago, Widdowson et al. recognized 2 M protein–related antigenic groups (I and II) in GAS that matched known skin and throat M serotypes (*7*). Skin types were subsequently found to have a cell surface lipoproteinase that binds fibronectin and causes opacity in horse serum (serum opacity factor [SOF]) (*8*). SOF is absent from identified rheumatogenic M types.

Using molecular techniques to differentiate M protein classes, Bessen et al. found that class I strains show a correlation with SOF-negative strains and contain serotypes associated with acute rheumatic fever (ARF) (*9*), whereas class II strains are associated with skin tropism. GCS/GGS only possesses class I M protein with a surface-exposed conserved region similar to M protein of known rheumatogenic GAS strains (*4*). Most human GCS/GGS appear to be SOF negative, although SOF-positive *emm* types (*stG166b*.*0* and *stG480*.*0*) have been reported (*10*). Although there are no published cases of ARF proven to have been caused by GCS/GGS, M protein characteristics of GCS/GGS probably play a role in clinical disease and tissue tropism, and suggest the potential for rheumatogenicity (*11*).

We conducted this study in Aboriginal communities of tropical Australia in which rates of ARF and rheumatic heart disease (RHD) are among the highest reported; however, in this region streptococcal pharyngitis is apparently rare and pyoderma is common (*12*). Outbreaks of acute poststreptococcal glomerulonephritis (APSGN) are also common (*13*). The primary aim of the study was to investigate the epidemiology of β-hemolytic streptococci and to determine whether there are unique aspects applicable to the pathogenesis of ARF/RHD. We also used molecular typing to specifically characterize the epidemiology of GCS/GGS throat carriage, pharyngitis, and skin infection in these communities and to examine their relationship to GAS epidemiology and ARF.

## Methods

### Community Surveillance

The study was conducted in 3 remote Aboriginal communities located in the northern part of Northern Territory in Australia in which the prevalence of RHD was >25 per 1,000 population compared with <1 per 1,000 in the non-Aboriginal population. community consultation, ethical approval (Human Research and Ethics Committee of the Northern Territory Department of Health and Community Services and Menzies School of Health Research, Darwin, Australia), household enrollment, data collection, and surveillance for ARF have been reported in detail (*12*). Surveillance was conducted in community 1 from July 2003 through June 2005, community 2 from July 2003 through June 2004, and in community 3 from July 2004 through June 2005. Local logistic problems restricted data collection in community 2. Community 1 is ≈500 km from community 2 and 700 km from community 3. The communities’ names have not been used at their request.

A high degree of day-to-day population mobility prevented regular follow-up of persons over an extended period. Households were representative of family groupings and were studied as distinct epidemiologic units. Study households were selected on the basis that at least 1 occupant had a known history of ARF or RHD; this was done to increase chances of encountering additional cases of ARF. A household was defined as a family group that lived in 1 house or 2 adjacent houses. Persons were considered to belong to a household if they said they belonged at enrollment and were present on at least 2 subsequent visits. Crowding was based on the number of occupants per bedroom.

We attempted to visit each household on a monthly basis. At each visit, all children and adults present were questioned about sore throat and skin sores. All throats were examined and swabbed for culture, limbs and exposed areas were examined, and pyoderma lesions were also swabbed. Each personal contact was called a consultation.

### Laboratory Methods

Specimen transportation, culture methods, and species identification have been described (*14*). Only large colony–forming β-hemolytic streptococci were selected and Lancefield grouped by using a Streptococcal Grouping Kit (Oxoid Diagnostic Reagents, Basingstoke, United Kingdom); suspected GAS isolates were tested for pyrrolidonyl arylamidase. Care was taken to exclude groups A, C, and G isolates of *S*. *anginosus* (*15*). Occasionally, *S*. *anginosus* morphology and β-hemolysis resembled that of GAS, but when colonies were streaked out and incubated overnight, the plates had a distinctive caramel odor. *S*. *anginosis* also failed to provide a PCR product for *emm* sequence typing.

The procedures for *emm* sequence typing followed those of the Centers for Disease Control and Prevention (CDC) (Atlanta, GA, USA) (*10*) with minor modifications. Seqman software (DNASTAR Inc., Madison, WI, USA) was used for sequence analysis and results were compared with the CDC *emm* sequence database. New *emm* sequence subtypes were assigned by the moderator. In this article, an *emm*ST refers to an *emm* sequence subtype. We also examined the translated *emm* sequences for plasminogen binding A repeats to identify *emm*STs of the plasminogen binding M-like protein (PAM) phenotype (*16*).

### Data Analysis

Epidemiologic data were analyzed by using Stata 8 (Stata Corporation, College Station, TX, USA). Confidence intervals were calculated by using standard methods. Correlation of household crowding and carriage was done by using Pearson correlation coefficient. Because of variability in household visits and the number of persons present at each visit, recovery rates were expressed per 100 consultations.

## Results

We enrolled 49 households and made 531 household visits. These households provided 4,841 throat swabs and 484 skin sore swabs from 420 episodes of pyoderma. Limited data were obtained from community 2, and most of the comparative analysis was done between communities 1 (population ≈2,500) and 3 (population ≈1,800). These communities are truly remote, being accessible only by air for much of the wet season (December to April).

We identified and *emm* sequence-typed 350 isolates of GAS, 80 isolates of GCS, and 257 of GGS (Table 1). Four new GCS/GGS nucleotide sequence subtypes were described (GenBank accession nos. in parentheses): *stC839*.*2* (AM403090), *stC1400*.*3* (AM403091) *stC1400*.*4* (AM403092), and *stG480*.*3* (AM403093). GGS was isolated from only 1 child with a sore throat; this isolate was *stG6792*.*0*, one of the most common types. GGS/GCS was recovered from persons with 9 episodes of pyoderma, but always with *Staphylococcus aureus*, and in 2 persons with GAS. There were 7 different *emm*STs identified in skin swabs; most belonged to common types.

**Table 1:** Streptococcal *emm* sequence subypes (STs) of study isolates by Lancefield type in 3 communities, Northern Territory, Australia*

Subtype	Community 1			Community 2			Community 3		Total	Total rate†
	No.	Rate†		No.	Rate†		No.	Rate†		
GCS *emm*ST										
stC839.0	29	0.96							29	0.60
stG643.0	26	0.86							26	0.54
stGrobn.0	9	0.30							9	0.19
stC6979.0	8	0.26					1	0.07	9	0.19
stC839.2	4	0.16							4	0.08
stC6746.0	1	0.03					1	0.07	2	0.04
stC2sk.1							1	0.07	1	0.02
Total	77	2.54		–	–		3	0.20	80	1.65
GGS *emm*ST										
*stC1400.0*	34	1.13		5	1.69		27	1.76	66	1.36
*stG4831.0*	34	1.13					7	0.46	41	0.85
*stG480.0*	28	0.93					10	0.65	38	0.78
*stG6792.0*	35	1.16		1	0.34		1	0.07	37	0.76
*stC74a.0*	25	0.83		1	0.34		3	0.20	29	0.60
*stC6979.0*	8	0.26					2	0.13	10	0.21
*stC5344.1*	9	0.30							9	0.19
*stG6.0*							6	0.39	6	0.12
*stG10.0*							5	0.33	5	0.10
*stG2078.0*							4	0.26	4	0.08
*stC.NSRT2.0*	1	0.03					2	0.13	3	0.06
*stC36.0*							3	0.20	3	0.06
*stC1400.4*							2	0.13	2	0.04
*stC1400.3*							1	0.07	1	0.02
*stG166b.0*							1	0.07	1	0.02
*stG480.3*							1	0.07	1	0.02
*stG652.0*	1	0.03							1	0.02
Total	175	5.76		7	2.37		75	4.90	257	5.30

*GCS, group C streptococci; GGS, group G streptococci. Lancefield type and *emm*STs do not always match and *stC6979* can be either GCC or GGC. †Per 100 consultations.

**Figure 1:**
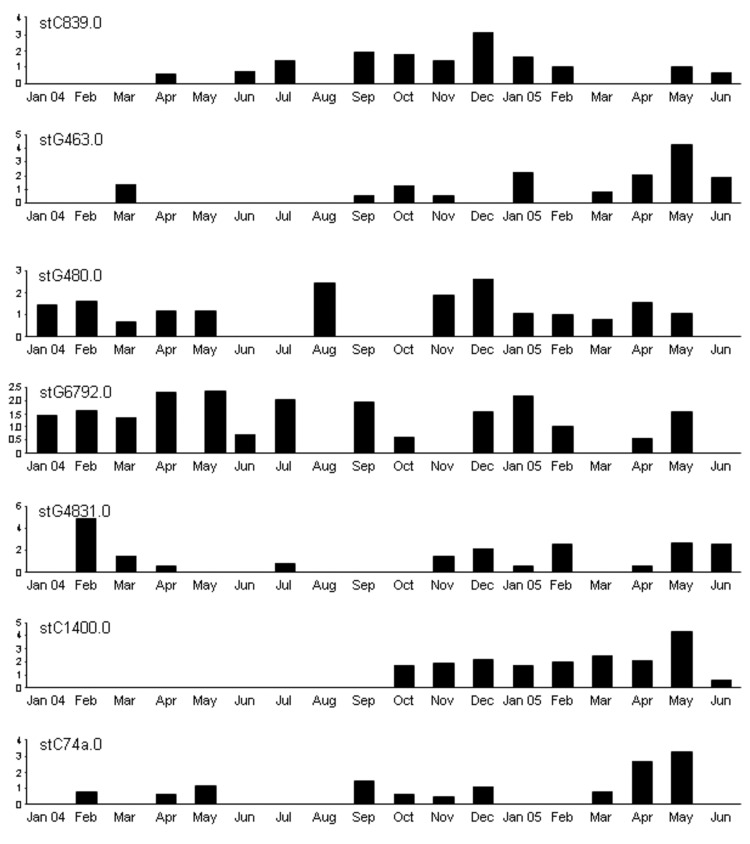
Monthly recovery rates of most common *Streptococcus dysgalactiae* subsp. *equisimilis* (group C and group G streptococci) *emm* sequence subtypes (STs) in community 1, Northern Territory, Australia. Values along the y-axes are no. bacterial isolates per 100 consultations. No obvious pattern of sequential strain replacement was seen as with *Streptococcus pyogenes* (group A streptococci) (*17*).

GCS were distributed unevenly (Table 1). The recovery rate for community 1 was 10 times that for community 3. Recovery rates for GGS and GAS across the communities were more even (*17*), which suggested that that the difference observed for GCS may be real. Throat swab samples from 154 (23.9%) children (those <15 years of age) had GCS/GGS, and samples from 126 (19.5%) children had GAS. Although GGS was more prevalent in community 1, there was greater diversity of *emm*STs in community 3, with 15 different *emm*STs compared with 9 different *emm*STs in community 1. Month-to-month recovery rates varied widely with medians of 3.3 (interquartile range [IQR] 1.2–3.3) per 100 consultations for GCS and 5.0 (IQR 3.4–7.5) for GGS in community 1, and 3.6 (IQR 1.6–5.9) for GGS in community 3. There was no apparent seasonal variation, although recovery rates of GCS, GGS, and GAS from throat swab samples peaked together in communities 1 and 3 during May 2005. A region-wide APSGN outbreak at that time was attributed to GAS *emm*55.0 (*18*), but recovery rates of GCS *stG643*.*0*, GGS *stC1400*.*0*, and GGS *stC74a*.*0* also increased sharply (Figure 1). This finding went largely unnoticed until *emm* typing of GCS/GGS was completed >12 months later.

The age distribution of GCS/GGS throat carriage in these communities was similar to that of GAS, with the highest recovery rates in 5- to 9-year-old children and 10- to 14-year-old children (*12*). However, different *emm*STs of GCS/GGS did not appear to cycle through the community in the same way as GAS (Figure 1) (*17*). There was no evidence of sequential *emm*ST replacement. It was difficult to follow persons month by month, although we did identify a child who had GCS *stC839*.*0* in throat samples on 11 occasions over 14 months and a household that harbored GGS *stG4831*.*0* for at least 18 months. GCS appeared to be highly prevalent (>5.0/100 consultations) in 3 households in community 1 yet was absent from 3 others in the same community. There was poor correlation between recovery rates of both GCS and GGS and household crowding (r = 0.24).

**Figure 2:**
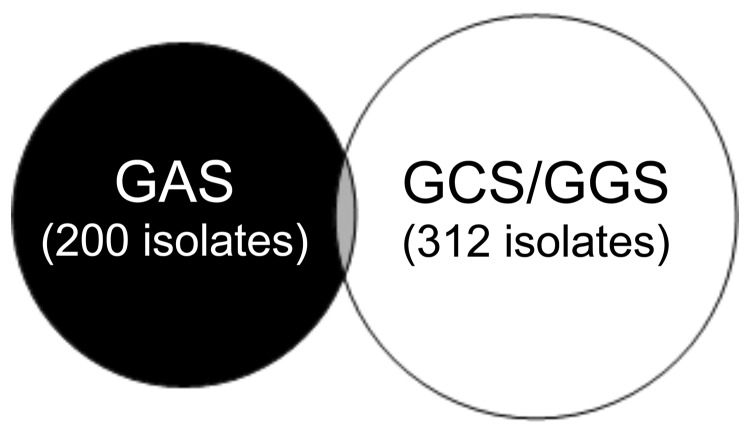
Venn diagram of positive throat swabs, Northern Territory, Australia, showing that group A streptococci (GAS) and *Streptococcus dysgalactiae* subsp. *equisimilis* (GCS/GGS) appear almost mutually exclusive. Thirteen persons had GAS and GCS or GGS, and 1 child had GAS, GCS, and GGS.

When GCS/GGS throat isolates were compared with GAS throat isolates, they appeared to be almost mutually exclusive (Figure 2); on only 14 occasions were GAS and GCS or GGS recovered together. The *emm*STs of the 15 isolates (1 child had GAS, GCS, and GGS) were representative of the whole GCS/GGS population (Table 2) and no *emm*ST was dominant. These results are consistent with the assumption that throat carriage of GAS and carriage of GCS/GGS are independent of each another. However, on further investigation, this did not appear to be true. Persons with a positive throat culture for GAS during the study were more likely to have a positive culture for GCS/GGS than those who never had GAS recovered from the throat (65 [36%] of 180, 95% confidence interval [CI] 22%–33% compared with 168 [17%] of 993, 95% CI 15%–19%, relative risk 2.3, 95% CI 1.7–3.0). The relative risk for persons who had >6 throat swabs taken over the course of the study and for those who had <6 swabs taken was the same. We observed a poor correlation between household recovery rates of GAS and GCS/GGS (r = 0.39 for community 1 and r = 0.16 for community 2).

**Table 2:** *Streptococcus dysgalactiae* subsp. *equisimilis* *emm* sequence subtypes (STs) of 15 isolates from 3 communities, Northern Territory, Australia*

Subtype	No. isolates
GCS *emm*ST	
*stC2sk.1*	1
*stC839.0*	2
*stGrobn.0*	1
GGS *emm*ST	
*stC-NSRT2.0*	1
*stC1400.0*	3
*stC1400.3*	1
*stC74a.0*	3
*stG480.0*	1
*st4831.0*	1
*stG6.0*	1

*Isolates obtained from throat swab samples that also contained *S*. *pyogenes*. GCS, group C streptococci; GGS, group G streptococci.

There was a marked discrepancy between β-hemolytic streptococcal recovery rates from the throat and the skin. GCS/GGS comprised 328 (60%) of 548 throat isolates (95% CI 56%–64%) and 9 (6.9%) of 131 skin isolates (95% CI 3.2%–12.6%) skin isolates. Sequence analysis of GCS/GGS *emm* showed that all isolates from throat and skin samples were negative for the skin-tropic determinant PAM.

Table 1 shows that 2 *emm*STs with group C carbohydrate by Lancefield typing had group G *emm* sequences (*stG643*.*0* and *stGrobn*.*0*) as determined by using the CDC database (*10*). Likewise, 8 GGS (*stC1400*.*0*, *stC74a.0*, *stC6979*.*0*, *stC5344*.*1*, *stCNSRT2*.*0*, *stC36*.*0*, *stC1400*.*3*, and *stC1400*.*4*) belonged to *emm*ST with GCS characteristics. Of 19 *stC6979*.*0* isolates, 9 were GCS and 10 were GGS. In May 2004, GCS *stC6979*.*0* and GGS *stC6979*.*0* were isolated from 2 persons in the same household. Initial investigation of the distribution of virulence genes in these isolates suggests that the GCS and GGS *stC6979*.*0* are 2 distinct strains. Further studies to differentiate them are under way.

## Discussion

The reported prevalence of GCS/GGS carriage and incidence of related disease varies greatly worldwide. Most studies originate from temporal climate regions of the Northern Hemisphere and limited data are available from tropical regions. In the minds of most researchers and clinicians, the contributory role of GCS/GGS to acute pharyngitis is consistent with supporting evidence from numerous studies, albeit of varying quality (*19**, **20*). Outbreaks of GCS/GGS-related disease have also been reported (*21*). However, several studies, some of good quality, are less supportive of this view (*22**,**23*). Our surveillance failed to produce convincing evidence of GAS pharyngitis in children of these communities (*12*) and GCS/GGS pharyngitis. We did show that GCS/GGS is more commonly found in the throat than GAS. Moreover, study participants who carried GAS at any time were more likely at some stage to carry GCS/GGS. The link is probably household environmental factors, but there may be a streptococcal carrier phenotype (*24*). There is no evidence from our data that GCS/GGS displaces GAS from the throat.

Reported rates for throat carriage of GCS ranged from 0% to 12% in 1 Finnish community (*25*) and from 0% to 9.3% in Indian schoolchildren (*26*). GGS throat carriage was more common than GAS in this study (*12*); this was also true for Indian and Bangladeshi schoolchildren (*26**,**27*) and a Nigerian community (*28*). However, results of studies conducted >10 years ago should be interpreted with caution because of previous taxonomic confusion with failure to distinguish small and large colony forms of GCG and GGS.

Throat carriage of GCS/GGS, as distinct from carriage of GAS, was quite uneven. GCS was concentrated in a few households in community 1 where long-term carriage was common, but GCS was nearly absent from community 3. The reason for this absence is unknown. GGS was more evenly distributed across the communities, but more highly concentrated in specific households. The community pattern of sequential strain replacement seen with GAS (*24**,**29*) was absent, which suggests that acquisition of M protein type–specific immunity against GCS/GGS may not play a role in these communities. There is no evidence that type-specific immunity is protective against GCS/GGS (*5**,**30*). In addition, we observed no seasonal variation of GAS carriage (*12*).

Although these isolates were recovered from persons in remote communities of Australia and new subtypes of established *emm* types were found, no new *emm* STs were found. Until now, there has been little published information regarding the existing scope of *emm* types of GCS/GGS. The findings of this study suggest that there may not be a huge diversity, at least not to the extent that is seen with GAS.

The degree of throat tropism of GCS/GGS and lack of independent skin pathogenicity was a conspicuous finding. GCS/GGS causes many diseases similar to GAS, including skin and soft tissue infection (*3*), but reports of childhood pyoderma are few. GGS was found in 3% of pyoderma lesions in an Indian study, but always with *S*. *aureus* (*31*). Similar rates were reported from children in Trinidad (*32*). A West African study reported a 16% recovery rate of GGS from pyoderma (*28*), but this study was conducted >35 years ago. APSGN has been associated with GCS/GGS pyoderma in Trinidad (*32*), although the evidence for causation is tenuous. The Top End outbreak of APSGN in May 2005 in the Northern Territory of Australia was not associated with an increase in GCS/GGS skin infection in study communities. However, an apparent but unexplained increase in throat carriage of GCS/GGS occurred during this period, concomitant with increased throat carriage and skin recovery of GAS (*17*).

Although GCS/GGS has class I M protein, other factors could account for the observed tropism. In GAS, PAM is associated with skin tropism and *emm* pattern type D (*33*). An animal model of pyoderma suggests that skin infection with these strains requires streptokinase and PAM-bound plasminogen (*34*). However, other mechanisms must be involved because pyoderma can also be caused by non-PAM pattern D types and other *emm* pattern types. The GCG/GGS isolates in this study, as elsewhere, lack PAM. The critical gene for streptokinase activity in GAS, *ska* (the subcluster 2b β-domain), may have been acquired from GCS/GGS by lateral gene transfer (*33*).

There is additional evidence for lateral gene transfer with *rofA* and related genes. The gene encoding the key determinant for GAS binding to skin fibroblast fibronectin, SfbI, is located in a highly recombinatorial region of the GAS genome (*35*). The s*fbI* gene has a homolog, g*fbA*, in GCS/GGS that is likely a product of horizontal gene transfer and recombination (*36*). The role of fibronectin binding in skin and soft tissue infection has yet to be elucidated. The *rofA* gene is a positive regulator of s*fbI* and is present in GAS *emm* patterns types A–C and E, but is less common in pattern type D. GAS *rofA* is another gene that was possibly acquired from GCS/GGS by horizontal gene transfer (*37*). GAS *emm* patterns A–C are more phylogenetically primitive and less genetically diverse (*38*) than *emm* pattern types D and E. The throat may have been the original niche for human colonization with GAS, and the ability to cause skin infection may be a more recently acquired trait.

Studies of bacterial housekeeping genes indicate that most gene traffic is toward GCS/GGS from GAS (*5*) and importation of GAS alleles into GCS/GGS is a relatively recent event. GAS is a completely human-adapted organism and human strains of GCS/GCS are more likely to be related to their animal flora origins. Humans may have acquired specific strains of GCS/GCS through animal domestication and these bacteria are now becoming human-adapted; 1 mechanism appears to be through phage-mediated acquisition of GAS alleles (*5*). There is some evidence that this process is more intense where the community streptococcal burden is high and strain turnover is rapid, such as in remote Aboriginal communities of the Northern Territory (*39*). If this is true, we could witness a regional increase in virulence of GCS/GGS over time, possibly including acquisition of rheumatogenic determinants.

Evidence is lacking that GCS/GGS causes ARF. Nonetheless, mouse antibodies to GCS/GGS M protein react with human cardiac myosin (*11*), and levels of antibodies to streptolysin O and hyaluronic acid increase after infection with GCS/GGS (*3*). ARF is driven by an exaggerated immune response to as-yet-undefined streptococcal epitopes, with possible immune priming from sequential streptococcal infections (*40*). An immune response to GCS/GGS, whether or not it is protective, may contribute to the priming process even if subsequent GAS infection is an absolute requirement for ARF.

We did not find a comparable study that examined the community and household dynamics of GCS/GGS carriage. Our study was originally intended as a longitudinal cohort study. However, the high population mobility, household turnover, and disruption of local community events compromised longitudinal surveillance (*12**,**17*). As such, the study became a series of point prevalence observations with accompanying data limitations. The study focused on households rather than persons, given the transience of the population, and we looked actively for throat and skin infection rather than waiting for presentation at the community health center. There were potential problems with variability of specimen collection and processing, and the lack of data from community 2 necessitated a move to community 3. We investigated selected study households, which may not have been representative of the whole community.

Because there is a relatively poor correlation between *emm* sequence type and GCS/GGS clone than with GAS (*5*), epidemiologic studies of GCS/GGS based on *emm* typing may need to be supplemented by techniques such as multilocus sequence typing, which define clonal type. Likewise, Lancefield grouping provides useful information, but is an unreliable epidemiologic tool unless supplemented by other methods. Nevertheless, the prospective nature of this study, and its size, make it likely that its findings provide a reasonable representation of the true epidemiology of GCS/GGS in this population. GCS/GGS was common in remote Aboriginal communities with high rates of streptococcal disease. Its contribution to illness, and even death, may manifest through indirect pathways, some of which have yet to be determined.
